# Effect of Bone Morphogenetic Protein-2 in the Treatment of Long Bone Non-Unions

**DOI:** 10.3390/jcm10194597

**Published:** 2021-10-06

**Authors:** Thomas Fuchs, Josef Stolberg-Stolberg, Philipp A. Michel, Patric Garcia, Susanne Amler, Dirk Wähnert, Michael J. Raschke

**Affiliations:** 1 Clinic for Orthopaedics, Trauma-, Hand- and Reconstructive Surgery, Vivantes Klinikum im Friedrichshain, Landsberger Allee 49, 10249 Berlin, Germany; Thomas.Fuchs@vivantes.de; 2 Department of Trauma-, Hand- and Reconstructive Surgery, Albert-Schweitzer-Campus 1, University Hospital Muenster, Building W1, 48149 Muenster, Germany; Philipp.Michel@ukmuenster.de (P.A.M.); Patricio.Garciacaso@ukmuenster.de (P.G.); michael.raschke@ukmuenster.de (M.J.R.); 3 Institute of Biostatistics and Clinical Research, University of Muenster, Schmeddingstrasse 56, 48149 Muenster, Germany; Susanne.Amler@ukmuenster.de; 4 Department of Trauma and Orthopedic Surgery, Protestant Hospital of Bethel Foundation, Campus Bielefeld-Bethel, University Hospital OWL of Bielefeld University, Burgsteig 13, 33617 Bielefeld, Germany; Dirk.Waehnert@evkb.de

**Keywords:** bone morphogenetic protein, long bone non-union, pseudarthrosis, fracture, bone healing

## Abstract

Background: Delayed fracture healing continues to cause significant patient morbidity and an economic burden to society. Biological stimulation of non-unions includes application of recombinant bone morphogenetic protein-2 (rhBMP-2). However, rhBMP-2 use continues to be a matter of controversy as literature shows scarce evidence for treatment effectiveness. Questions: The objective of this study was to evaluate the effectiveness of rhBMP-2 treatment on long bone non-unions measuring union rate and time to union. Furthermore, we assess risk factors for treatment failure. Methods and patients: A total of 91 patients with non-unions of long bones were treated with rhBMP-2 (*n* = 72) or standard care without BMP (*n* = 19) at our institution. Patient characteristics, comorbidities, nicotine consumption, and complications were recorded. Bone healing was assessed by plane X-rays and clinical examination. Patients were followed up with for 24 months. Results: Overall, there was significantly faster bone healing after rhBMP-2 application compared to the no-BMP group (*p* < 0.001; HR = 2.78; 95% CI 1.4–5.6). Union rates differed significantly between rhBMP-2 compared to the no-BMP group (89% vs. 47%; *p* < 0.001). At the humerus, there was neither a significantly higher union rate in the rhBMP-2 (83%) compared to the no-BMP group (50%) (*p* = 0.26; *n* = 12) nor a faster bone healing with a median time of 9 months in both groups (HR = 2.01; 95% CI 0.49–8.61; *p* = 0.315). The 33 femora treated using rhBMP-2 healed significantly faster than 9 femora in the no-BMP group (HR = 2.93; 95% CI 1.00–8.4; *p* = 0.023) with significant differences in union rate with 85% and 44%, respectively (*p* = 0.022). Regarding tibia non-unions, 25 out of 27 (93%) healed with a median of 9 months after rhBMP-2 application with no significant difference in the no-BMP group (33%) in time to union (*p* = 0.097) but a significantly higher union rate (*p* = 0.039). There was no effect of comorbidities, age, sex, soft tissue damage, or nicotine use on time to union, union rate, or secondary interventions. Conclusion: Consistent with the literature, overall, significantly higher union rates with reduced time to union were achieved after rhBMP-2 application. Femoral and tibial non-unions in particular seem to profit from rhBMP-2 application.

## 1. Introduction

Fracture healing and restoration of bone to its pre-injury composition and function is a biologically optimized process [1]. Nevertheless, around 10% of all fractures show impaired healing. Clinically, open tibia fractures pose a particular challenge to surgeons as complication rates up to 50% have been reported [2]. Patients are exposed to significant restrictions to their daily life and substantial costs to the health care systems are generated [3]. Bone healing can be affected by inadequate reduction, impaired biology, mechanical instability, infection, comorbidities of the patient, and the extent of soft tissue damage [4].

Standard treatments of non-unions include mechanical stabilization and biological stimulation. Autologous cancellous bone grafting is still the gold standard to encourage bone union. However, there are limitations such as restricted availability, donor site morbidities, blood loss, and increased operation and hospitalization time [5,6]. Alternatively, new techniques such as the reamer irrigator aspirator (RIA) (DePuy Synthes, Zuchwil, Switzerland) have been introduced but also represent an invasive procedure with side effects such as fat embolism, thermal necrosis, and fractures at the harvest site [7]. Further options such as segmental bone resection and bone transport distraction, nail dynamization, and high energy extra-corporal shock wave therapy are subject to special indications. Therefore, the development and clinical evaluation of less invasive treatment options have become inevitable.

Bone morphogenetic proteins (BMPs) as powerful osteoinductive agents has gained the attention of the scientific community as well as surgeons facing the problems of non-unions since 1970 [8]. BMPs belong to the TGF-β superfamily and are characterized by their sequence and function [8]. Nearly 20 structurally related BMPs have been found. So far, rhBMP-2 has received approval for the treatment of open tibia shaft fractures after fracture reduction and intramedullary un-reamed nail fixation, sinus or local alveolar ridge augmentation, and spinal fusion by the US Food and Drug Administration. Off-label use has shown good results in posterior lumbar and cervical spine fusion [9]. Although there are multiple animal models showing the beneficial effect of BMPs in long bone non-unions, few studies analyze the clinical effectiveness [10]. Some clinical trials only involve small sample populations and lack control groups [11,12].

Considering the high costs of BMP treatment and the paucity of evidence for treatment effectiveness, fracture region, type, and risk factors have to be identified to justify further usage [12]. The aim of this study is to compare non-unions in the long bones humerus, femur, and tibia with and without BMP treatment. Furthermore, we want to identify complications and risk factors on union-rate and time to union.

## 2. Patients and Methods

### 2.1. Study Design

The present study was a single-center, prospective, open-label study. It was conducted in our hospital and approved by the ethical commission (Ethik-Komission der Ärztekammer Westfalen Lippe; reference number 3100N7-212-WW; 3100N7-211-WW; 3100N7-210-WW; ClinicalTrials.gov identifier: NCT05065684).

**Figure 1:**
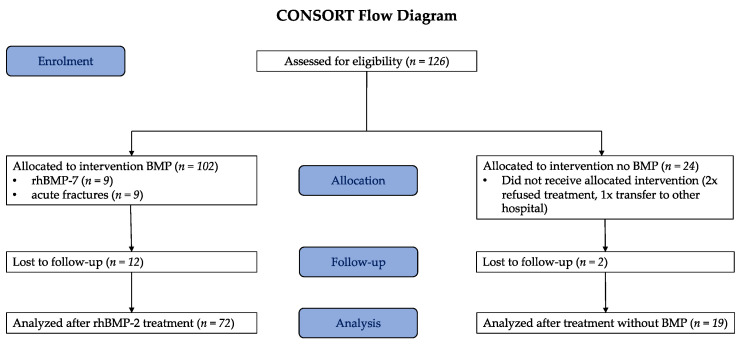
Consort flow diagram indicating enrolment, intervention allocation, and follow up.

Patients with clinical signs of infection at the side of non-union in combination with elevated CRP and leucocyte count, underage patients, cancer patients, refusal of study participation, and acute fractures were excluded. Standard care was defined as non-union resection and autograft of cancellous bone from the iliac crest. All patients were assigned for additional BMP treatment. However, 19 patients did not receive BMP due to delayed insurance confirmation for BMP reimbursement. All procedures were performed after informed consent of the patient. Finally, 72 patients with long-bone non-unions were treated with rhBMP-2 and 19 patients received standard care without BMP (Table 1, Figure 1).

Fracture healing was assessed by radiological and clinical examination every three months. Clinically, the absence of pain on loading and the absence of abnormal movement at the non-union site were crucial. Radiologically, the presence of bridging callus on three of the four cortices was necessary in two plane X-rays to define the non-union as healed. Clinical follow-up was conducted by surgeons of our department that were partly involved in the procedure of BMP implantation. Radiological analysis was independently done by a radiologist.

### 2.2. Patient Demographics

A total of 126 patients were assessed for eligibility. Two patients were not included due to refusal to participate in this study. One patient was transferred to another clinic because of severe comorbidities, 9 patients were treated for an acute fracture, and 9 patients were treated with rhBMP-7. A total of 105 patients were treated at our institution between 2005 and 2011. Of them, 14 patients (12 from the rhBMP-2 and 2 from the no-BMP group) were lost during follow up and 91 (87%) were followed up for 24 months. In total, 50 male (55%) and 41 female (45%) patients were included (Table 2).

The mean age of the patients in the rhBMP-2 group was 52 years (minimum 21, maximum 81 years). Of them, 41 patients were male, 31 were female. In the sample, 33% of the fractures (24 of 72) showed open soft tissue damage. Severity was described following the classification of Tscherne and Oestern [13]: in 7 cases a grade 1, in 8 a grade 2, and in 9 a grade 3 open fracture were found. The localization of BMP application was 6 times the humerus, 33 times the femur, 27 times the tibia, 3 times the forearm, 2 times the upper ankle joint, and in 1 case the pelvis (os ilium). One out of six humerus non-unions occurred after one III° open fracture, the remaining 5 after closed fractures. In total, 33 femur non-unions were treated after a closed fracture in 23 cases, I° open fracture in 3 cases, II° open fracture in 4 cases, and III° open fracture in 3 cases. From 27 tibia non-unions, 16 after closed, 4 after I° open, 3 after II° open, and 4 after III° open fractures were treated.

In the no-BMP group, the mean age was 47 years (minimum 19, maximum 81 years); 9 patients were male, 10 were female. Of the 18 fractures, 4 (22%) were open fractures classified following Tscherne and Oestern as grade 1 in 1 case, grade 2 in 2 cases, and grade 3 in 1 case. Anatomical location (Table 1) was 6 times the humerus, 9 times the femur, 3 times the tibia, and 1 time the forearm. Within the group of 6 humerus fractures, 5 non-unions after closed fracture and 1 non-union after I° open fracture were treated without BMPs. There were 8 non-unions after former closed fractures and 1 non-union after II° open femur fracture. Regarding the tibia, there were 3 fractures, of which 2 non-unions occurred after closed and 1 non-union after III° open fractures were additionally analyzed in the no-BMP group. In all cases, the indication for BMP use was a non-union as defined by a minimum of insufficient bone healing 6 months after fracture.

### 2.3. Preparation and Implantation of rhBMP-2

RhBMP 2 InductOs^®^ (Medtronic, Watford, Hertfordshire, UK) was used. InductOs^®^/rhBMP 2 contains 12 mg dibotermin alfa and was used in 72 of the presented cases. In all cases, the fracture site/site of none-union was debrided, wound irrigation was conducted, hemostasis was achieved, osteosynthesis was finished, and iliac crest autograft was added. RhBMP-2 was then prepared according to manufacturer’s instruction. Briefly, enclosed solvent was added to rhBMP-2 and gently swirled. The matrix was opened; 8 mL solution was equally distributed and left within the tray. After 15 min, the entire sponge was applied to the fracture site. The entire volume was immediately placed at the site of pseudarthrosis. Complete soft tissue coverage succeeded shortly in all cases. All patients were operated on by an experienced orthopedic surgeon of our department.

Clinically relevant retrospective data regarding medical history, co-morbidity, the localization, and the outcome was collected. Comorbidities were listed and categorized as follows: cardiovascular (peripheral arterial disease, hyperuricemia, coronary artery disease, bleeding disorders, hemophilia A, thrombocytopenia, blood loss anemia), metabolic (diabetes, adiposities, hypertonia, metabolic syndrome, liver disorders, alcoholic liver cirrhosis, alcohol abuse, hypothyroidism, hyperkalemia), neurologic (Parkinson, borderline personality, suicidality, epilepsy, drug abuse, sleep apnea syndrome, status post apoplexy), rheumatologic and allergic (atopic eczema, rheumatoid arthritis, bronchial asthma, chronic bronchitis), and infectious (MRSA, hepatitis A, B, and C). Among the BMP group, 13 patients showed active nicotine use over the period of treatment as compared to two in the no BMP group.

Statistical analyses were performed using IBM SPSS^®^ Statistics 22 for Windows (IBM Corporation, Somers, NY, USA). Comparison of time to union between BMP and no-BMP group was done using the Logrank test. Reverse Kaplan–Meier curves were plotted to illustrate cumulative incidence of time to union [14]. Associations between the union-rate as a binary outcome variable up to 24 months after treatment and different clinically relevant parameters were calculated by Fisher’s exact test [15]. Descriptive statistics were used for a more comprehensive presentation of the results of our prospective case series. Multivariable Cox regression analysis was used to adjust for potential confounders (i.e., comorbidities, age, sex, open fracture, nicotine use, body site) [16].

## 3. Results

**Figure 2:**
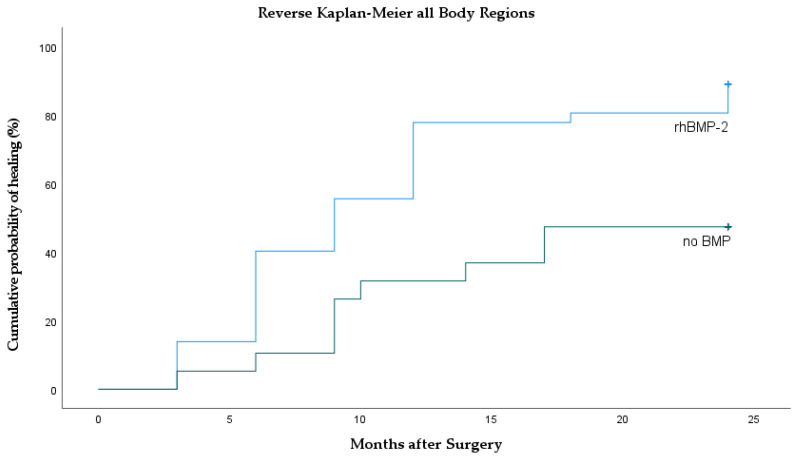
Reverse Kaplan–Meier curves of all body regions showing the incidence of bone healing with and without rhBMP-2 application.

In total, 91 patients were followed up for 24 months between 2005 and 2011. Overall, there was significantly faster bone healing after BMP application compared to the no BMP group (*p* = 0.001). Median time to bone union in the rhBMP-2 group was 9 months (95% CI 6.7–11.3) and significantly faster compared to the no-BMP group (median not reached; *p* < 0.001; HR = 2.78; 95% CI 1.4–5.6). In the no-BMP treatment group, 10 patients showed a persistent pseudarthrosis after 24 months; 3 times humerus, 5 times femur, and 2 times tibia were counted. Union rates differed significantly between rhBMP-2 compared to the no-BMP group (89% vs. 47%; *p* < 0.001; Figure 2.

**Figure 3:**
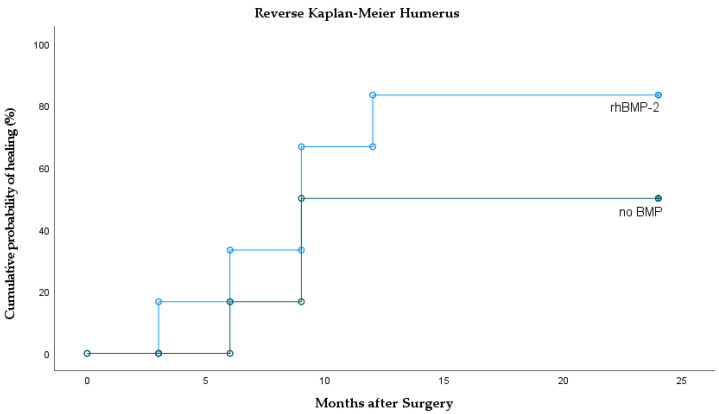
Reverse Kaplan–Meier curves of the humerus showing the incidence of bone healing with and without rhBMP-2 application.

Comparing the 12 treated humeri (Figure 3, Table 1), healing was achieved in 83% after rhBMP-2 compared to 50% without BMP treatment (*p* = 0.26). Median time to union was the same in the rhBMP-2 group (9 months) as compared to the no-BMP group (median 9 months; HR rhBMP-2 vs. no-BMP = 2.1; 95% CI 0.49–8.61; *p* = 0.315).

**Figure 4:**
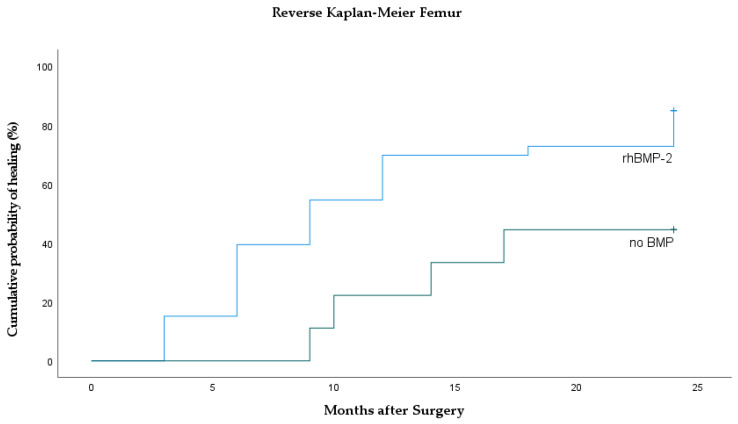
Reverse Kaplan–Meier curves of the femur showing cumulative incidence of bone healing separately for rhBMP-2 and control (no BMP).

Out of 42 treated femora (Figure 4), bone healing was achieved in 85% in the rhBMP-2 group compared to 44% in the no-BMP group (*p* = 0.022). Median time to union was significantly faster in the rhBMP-2 group (9 months). Within the no-BMP group, the median time to union had not been reached at the date of the analysis (HR rhBMP-2 vs. no-BMP = 2.93; 95% CI 1.00–8.4; *p* = 0.023).

**Figure 5:**
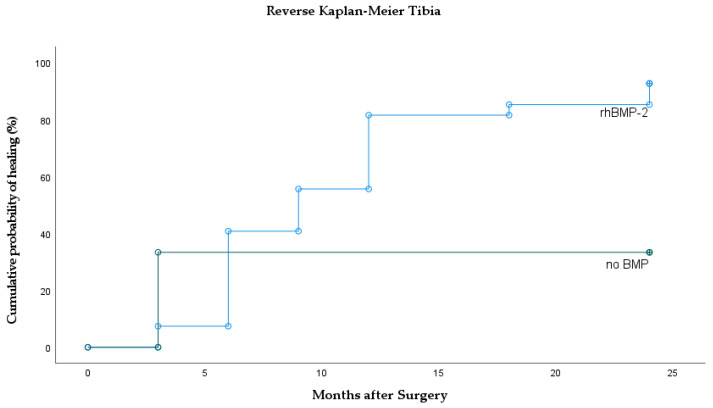
Reverse Kaplan–Meier curves of the tibia showing cumulative incidence of bone healing separately for rhBMP-2 and control (no BMP).

Out of 27 prior tibia non-unions, 25 healed after rhBMP-2 application. One prior non-union only showed partial healing and one showed no signs of consolidation at all. Out of 3 tibia non-unions that were treated without BMPs, only 1 healed after 3 months and 2 did not show signs of bone healing after 24 months (Figure 5). Within the tibia group, no significant differences were found regarding time to union (*p* = 0.097). In the rhBMP-2 group, median time to union was 9 months. There were no significant differences compared to the control (median not reached; *p* = 0.50; HR = 2.0). However, rhBMP-2 had a significantly higher union rate compared to the control group (93% vs. 33%; *p* = 0.039). Summarizing BMP application at the tibia, there was 93% after prior non-union and rhBMP-2 application and 33% in the no BMP group.

Secondary interventions were necessary in 17 (18.7%) of all cases. After rhBMP-2 use, 14 revisions (3 × repeated wound irrigation, 3 × implant revision, 2 × dynamization, 4 × repeated cancellous bone application, 2 × repeated rhBMP-2 application) accounted for 19.5%, of which 6 (22.2%) occurred after treatment of tibia non-unions. Comparable amounts of complication were recorded in the no-BMP group with 3 (15.8%).

Multivariable Cox regression analysis showed no meaningful effects of the single factor comorbidities (6 × cardiovascular, 22 × metabolic, 7 × neurologic, 7 × rheumatic/ allergic, 2 × infectious) nicotine abuse (22 ×), body region, secondary interventions, age, or sex on time to bone union. Additionally, there was no effect of open fracture compared to closed fracture on time to bone healing.

## 4. Discussion

The objective of this study was to characterize bone healing in multiple body regions after BMP application, analyze risk factors for time to union and union rate, and complications, and compare them with a no-BMP group.

Time to bone union after BMP application varies within literature and only some authors report shortened time spans. Govender et al. describe significantly faster bone healing after application of rhBMP-2 on open tibia shaft fractures [17]. Similar durations are reported in additional studies [18,19,20]. In contrast, significantly accelerated time to union could not be observed by other authors [21,22]. In our own observation, overall median bone healing after rhBMP-2 application of all bones was significantly faster compared to our control group (Table 1, Figure 2). Comparing only tibia non-unions after rhBMP-2 application, there was no difference in time to union (Figure 5). Thus, our data favor the extended use of rhBMP-2 but, inconsistent with the literature, tibia fractures do not seem to heal faster.

In the literature, there is evidence for higher union rates after acute tibia fractures and BMP application [17,19,20]. Prior non-unions responded heterogeneously on BMP treatment [17,23,24]. Friedlaender et al. showed in a prospective, randomized, multi-center study rhBMP-7 to be safe and effective in 124 tibial non-unions but no significant difference was found in comparison to autologeous bone [24]. Equally, Geesink et al. were not able to show a significant difference treating critical size fibular defects with demineralized bone or rhBMP-7 [25]. Calori et al. were only able to show superior results in comparison to platelet rich plasma treatment [18]. Our experience in this study shows significantly higher union rates after rhBMP-2 application at the tibia and femur. However, it has to be stated that only 9 patients were included into the femur and only 3 patients in the tibia control group.

Fracture healing in this study was defined by plain X-rays and clinical examination. This is consistent with other studies and seems to be the gold standard [17,19,20]. However, it is known that there is a lack of consensus on fracture healing based on X-rays amongst orthopedic surgeons [26]. Furthermore, clinical assessment depends greatly on the examiner’s technique and experience [27]. Hence, comparing union times and rates of various studies might not be legitimate. There might even be significant inaccuracy within each study owing to diagnostic limitations.

Secondary interventions to achieve bone union might be reduced by BMP application particularly at the tibia [18,24]. Govender et al. describe a reduction of 44% after open tibia fracture [17]. Other anatomic sites do not favor the use of BMP [21]. We could not find more secondary interventions after BMP application in any of our other groups. However, postoperative swelling and redness were regularly observed, particularly after rhBMP-2 application [27].

Infections are a regular event after surgery but do not seem to be affected by BMP implantation. Friedlaender et al. reported 14 infections in the BMP and 12 infections in the autograft group after treating 124 tibia non-unions [24]. Jones et al. described three infections requiring surgical revision after the treatment of 30 tibia fractures, one in the BMP group and two in the autograft group. However, it has to be stated that all infections occurred in Gustilo–Anderson type-III injuries [19]. A strong association between soft tissue damage and postoperative infection in all groups was also seen by Govender et al. [17]. In our study, all infections that caused repeated wound irrigation were originally open fractures but statistically there seems to be no significant effect. This might be caused by our small control group.

Hardware failure occurs in significantly fewer cases after BMP treatment of tibia fractures [17,19]. There is one study reporting osteolysis, significantly slower and impaired bone healing in the BMP group [22]. In our own study, 3 patients in the rhBMP-2 group needed implant revision and 2 tibia nails needed to be dynamized. Comparable rates of secondary interventions were found in our no-BMP group (15.8%). Further complications include ectopic ossifications induced by rhBMP-2 treatment which did not cause major complications or secondary surgery in our study.

In spine surgery patients with diabetes, hypothyroidism, and even smokers, a union rate of 95% was achieved. There was no significant difference to the healthy control group [28,29]. In the present study, we did not find significantly inferior outcomes in our patient groups with risk factors.

Economic evaluation and cost effectiveness of BMP application can only be carefully carried out as calculations depend on local clinical practice, care, and prices. However, there is one cost-utility analysis for BMP use in open tibia fractures. Here, an incremental cost per quality-adjusted life-year gained was calculated to be GBP 32,603. Thus, the effectiveness of additional BMP use might be improved if BMP prices are reduced and if it is used in severe cases only [30]. Another study states that the cost effectiveness of rhBMP-7, autograft, and Ilizarov fixateur are similar in the UK but favor conventional care over BMP use in Germany [12].

Limitations of this study are the single center-based data collection and inhomogeneous cohorts of patients. Additionally, comparison of outcomes between different body regions might not be legitimate as mechanical stabilization and bone biology can differ and the count is disproportionate. As in all other reports, definition of bone union by plain X-rays is insufficient and a CT scan would improve the quality of the study. Clinical assessment was also conducted but there was no quantifiable outcome score. Furthermore, there was no randomized group allocation.

In future, the use of BMPs will remain a matter of controversy. Recently raised concerns about the potential to induce malignancies have not been confirmed until now [31]. There is a research focus on biomaterial carriers which might improve pharmacokinetics [32]. Engineered bone tissue might be a promising field for further BMP application. However, the optimal cocktail of BMPs and other growth factors still has to be found to reproduce in vivo bone in the laboratory [33,34]. Additional areas of BMP applications such as gene therapy using vectors or BMP treatment of posttraumatic femoral head necrosis are still at the stage of pre-clinical research [35].

Concluding, the present study underlines the effective use of rhBMP-2 for the treatment long bone non-unions. However, other clinical studies show mixed evidence. Hence, future research needs to focus on clinical studies analyzing only specific subgroups [36,37,38].

## Figures and Tables

**Table 1:** Total number of non-unions (bone unions, union rate in %), and time to union for humerus, femur, and tibia, as well as degree of open fracture characterizing the study groups.

	No BMP	RhBMP-2
Humerus		
Closed	5 (2 = 40%)	5 (4 = 80%)
I° open	1 (1 = 100%)	
II° open		
III° open		1 (1 = 100%)
Number of Patients	6 (3 = 50%)	6 (5 = 83%)
Median time to Bone Union (months)	9	9
Femur		
Closed	8 (3 = 43%)	23 (20 = 83%)
I° open		3 (2 = 66%)
II° open	1 (1 = 100%)	4 (3 = 75%)
III° open		3 (3 = 100%)
Number of Patients	9 (4 = 44%)	33 (28 = 85%)
Median time to Bone Union (months)	-	9
Tibia		
Closed	2 (0 = 0%)	16 (15 = 94%)
I° open		4 (4 = 100%)
II° open		3 (2 = 67%)
III° open	1 (1 = 100%)	4 (4 = 100%)
Number of Patients	3 (1 = 33%)	27 (25 = 93%)
Median time to Bone Union (months)	-	9

**Table 2:** Patient characteristics including age, sex, prior treatment, treatment group, time to union, and comorbidities (LPF—locked plate fixation; IN—intramedullary nailing; C—conservative; EF—external fixation).

								Comorbidities				
Group	Sex	Age	Bone	Prior Treatment	Fracture	Time to union	Smoking	Cardiovascular	Metabolic	Neurologic	Rheumatologic	Infectious
rhBMP-2	m	31	Forearm	LPF	II° open	3						
rhBMP-2	m	23	Femur	LPF	II° open	6						
rhBMP-2	m	77	Femur	LPF	closed	3				1		
rhBMP-2	w	81	Femur	LPF	closed	3				1		
rhBMP-2	m	22	Femur	IN	II° open	3					1	
rhBMP-2	w	56	Femur	IN	closed	3						
rhBMP-2	w	41	Femur	IN	closed	6						
rhBMP-2	m	51	Femur	LPF	closed	Persistent non-union	Yes		1			
rhBMP-2	m	56	Tibia	IN	I° open	12	Yes					
rhBMP-2	w	54	Tibia	EF	closed	9				1		
rhBMP-2	w	40	Tibia	EF	III° open	9					1	
rhBMP-2	w	45	Tibia	IN	closed	18						
rhBMP-2	m	69	Femur	LPF	closed	Persistent non-union			1			
rhBMP-2	m	29	Tibia	IN	closed	6						
rhBMP-2	w	54	Femur	IN	III° open	12						
rhBMP-2	w	72	Femur	LPF	closed	12						
rhBMP-2	m	49	Femur	IN	II° open	Persistent non-union						
rhBMP-2	m	47	Tibia	LPF	closed	6						
rhBMP-2	w	56	Femur	LPF	closed	6						
rhBMP-2	m	54	Tibia	LPF	closed	6						
rhBMP-2	m	41	Tibia	LPF	closed	6						
rhBMP-2	w	74	Tibia	LPF	II° open	12						
rhBMP-2	m	52	Upper ankle joint	LPF	closed	12	Yes					
rhBMP-2	w	45	Tibia	IN	III° open	12						
rhBMP-2	w	49	Femur	IN	I° open	24						
rhBMP-2	m	47	Tibia	IN	I° open	9	Yes					
rhBMP-2	m	46	Femur	IN	closed	12	Yes					
rhBMP-2	m	37	Tibia	IN	closed	6						
rhBMP-2	w	41	Tibia	IN	closed	24	Yes	1				
rhBMP-2	m	51	Femur	IN	I° open	6						
rhBMP-2	m	52	Tibia	LPF	I° open	24						1
rhBMP-2	m	45	Tibia	LPF	closed	6	Yes					
rhBMP-2	m	21	Tibia	LPF	closed	12	Yes					
rhBMP-2	w	23	Tibia	IN	closed	6						
rhBMP-2	m	43	Humerus	LPF	III° open	9						
rhBMP-2	w	40	Femur	LPF	III° open	6					1	
rhBMP-2	m	60	Femur	IN	closed	9			1			
rhBMP-2	m	54	Femur	IN	closed	24						
rhBMP-2	m	57	Tibia	LPF	closed	Persistent non-union			2			
rhBMP-2	w	55	Tibia	LPF	III° open	12					1	
rhBMP-2	m	53	Femur	LPF	I° open	18		1				
rhBMP-2	m	27	Femur	IN	III° open	24						
rhBMP-2	w	80	Femur	LPF	closed	24			1			
rhBMP-2	w	51	Tibia	LPF	closed	6	Yes					
rhBMP-2	m	50	Tibia	IN	III° open	3			1			
rhBMP-2	m	48	Femur	IN	closed	9					1	
rhBMP-2	w	86	Tibia	C	closed	3			1			
rhBMP-2	m	71	Forearm	LPF	closed	12						
rhBMP-2	m	64	Humerus	IN	closed	9			1			
rhBMP-2	m	59	Femur	LPF	closed	3						
rhBMP-2	m	48	Femur	LPF	closed	9						
rhBMP-2	w	66	Os ilium	C	closed	3	Yes					
rhBMP-2	w	27	Tibia	IN	closed	12	Yes		1			
rhBMP-2	w	79	Humerus	C	closed	12			1			
rhBMP-2	w	73	Femur	IN	closed	9			1			
rhBMP-2	m	30	Upper ankle joint	LPF	closed	6	Yes				1	
rhBMP-2	m	84	Femur	LPF	closed	12			1			
rhBMP-2	m	40	Forearm	LPF	III° open	12						
rhBMP-2	w	59	Humerus	IN	closed	6			1			
rhBMP-2	w	61	Tibia	LPF	I° open	12						
rhBMP-2	m	29	Humerus	LPF	closed	3	Yes					
rhBMP-2	w	62	Femur	LPF	closed	6						
rhBMP-2	m	59	Femur	IN	closed	9						
rhBMP-2	w	55	Femur	IN	closed	12						
rhBMP-2	w	47	Tibia	IN	closed	9						
rhBMP-2	w	29	Femur	LPF	closed	Persistent non-union						
rhBMP-2	m	61	Femur	LPF	II° open	6						
rhBMP-2	m	52	Tibia	IN	II° open	Persistent non-union						
rhBMP-2	w	58	Humerus	LPF	closed	Persistent non-union						
rhBMP-2	m	43	Femur	C	closed	Persistent non-union						
rhBMP-2	w	80	Femur	LPF	closed	6			1			
rhBMP-2	m	52	Tibia	IN	II° open	6						
no BMP	m	81	Femur	LPF	closed	Persistent non-union		1	1			
no BMP	m	40	Forearm	LPF	II° open	17	Yes					
no BMP	w	73	Humerus	IN	closed	Persistent non-union						
no BMP	m	52	Tibia	IN	closed	Persistent non-union						
no BMP	m	19	Femur	IN	II° open	10				1		
no BMP	w	49	Femur	LPF	closed	Persistent non-union						
no BMP	w	62	Femur	IN	closed	Persistent non-union						
no BMP	m	46	Tibia	LPF	III° open	3	Yes		1	1		
no BMP	m	39	Humerus	LPF	closed	Persistent non-union			1			
no BMP	m	49	Humerus	IN	closed	Persistent non-union						
no BMP	w	69	Femur	LPF	closed	Persistent non-union						
no BMP	w	75	Femur	LISS	closed	14						
no BMP	w	62	Humerus	LPF	closed	6			1			
no BMP	w	58	Femur	IN	closed	17			1			
no BMP	m	47	Humerus	LPF	I° open	9		1	1	1		
no BMP	w	20	Humerus	IN	closed	9						
no BMP	w	20	Femur	IN	closed	Persistent non-union						
no BMP	m	21	Femur	IN	closed	9						
no BMP	w	31	Tibia	IN	closed	Persistent non-union				1		

## Data Availability

Upon contact to the corresponding author.
